# Alterungsbedingte Gefäßveränderungen am Beispiel der Arteria carotis

**DOI:** 10.1007/s00772-022-00901-5

**Published:** 2022-06-24

**Authors:** Benedikt Reutersberg, Philip Düppers, Anna-Leonie Menges, Claudia Schrimpf, Alexander Zimmermann, Jaroslav Pelisek

**Affiliations:** Klinik für Gefäßchirurgie, Universitätsspital Zürich, Rämistr. 100, 8091 Zürich, Schweiz

**Keywords:** Atherosklerose, Karotisstenose, Plaquemorphologie, Schlaganfall, Epigenetik, Atherosclerosis, Carotid stenosis, Plaque morphology, Stroke, Epigenetics

## Abstract

Einer der Hauptrisikofaktoren für das Vorliegen einer Karotisstenose und des karotisbedingten Schlaganfalls ist das Lebensalter. Ziel dieses Übersichtsartikels ist die Darstellung des aktuellen Wissensstands über altersbedingte Veränderungen der Gefäße am Beispiel der Karotisstenose.

Die Gefäßalterung (vaskuläre Seneszenz) als Abnahme struktureller und funktioneller Eigenschaften der Gefäßwand spielt sich auf verschiedenen Ebenen ab. Auf multizellulärer Ebene kommt es mit zunehmendem Alter hauptsächlich aufgrund von atherosklerotischen Veränderungen der Gefäßwand zu einer Zunahme von Gefäßvolumen und -durchmesser sowie der Intima-Media-Dicke. Auf zellulärer und extrazellulärer Ebene kommt es zur Abnahme von Elastinfasern, glatten Muskelzellen und der Gesamtzellularität sowie zur Zunahme der Lipid‑, Cholesterin- und Kalziumphosphatablagerungen und der Neovaskularisierung. Ursachen der Gefäßalterung auf molekularer Ebene sind insbesondere oxidativer Stress, chronische Entzündungsreaktion, mitochondriale Dysfunktion, epigenetische Veränderungen, Dysregulation der Expression nicht kodierender RNAs (ncRNAs) und die Zunahme der Seneszenz. Der altersbedingte Verlust der Heilungs- und Reparaturfähigkeit des Gewebes macht die Plaques vulnerabler und im Falle der A. carotis anfälliger für ischämische Schlaganfälle.

Zunehmende Erkenntnisse über den Einfluss des Alterns auf die Epigenetik und der ncRNAs in atherosklerotischen Plaques kann zukünftig das individuelle Risiko von Patienten genauer quantifizieren und zur Entwicklung zielgerichteter Therapiestrategien beitragen. Weitere Studien sind auf diesem Gebiet jedoch notwendig, um das gesamte Ausmaß der Gefäßalterung und den damit einhergehenden Erkrankungen zu verstehen, damit diesen dann gezielt entgegenwirkt werden kann.

## Abstract (en)

One of the main risk factors for the presence of carotid stenosis and carotid-related stroke is age. The aim of this review article is to present the current state of knowledge on age-related vascular changes using carotid stenosis as an example.

Vascular aging (vascular senescence) is a decrease of structural and functional properties of the vessel wall that takes place on different levels. At the multicellular level an increase in vessel volume and diameter as well as intima media thickness occurs with age mainly due to atherosclerotic changes in the vessel wall. At the cellular and extracellular levels there is a decrease in elastin fibers, smooth muscle cells, and total cellularity, an increase in lipid, cholesterol, and calcium phosphate deposition as well as neovascularization. The causes of vascular aging at the molecular level include, in particular oxidative stress, chronic inflammatory response, mitochondrial dysfunction, epigenetic changes, dysregulation of the expression of non-coding RNAs (ncRNAs), and the increase in senescence. Age-related loss of tissue healing and repair capacity make plaques more vulnerable and, in the case of the carotid artery, more susceptible to ischemic stroke.

Increasing knowledge of the influence of aging on the epigenetics and ncRNAs in atherosclerotic plaques can in the future more accurately quantify individual patient risk and contribute to the development of targeted therapeutic strategies; however, further studies are needed in this field to understand the full extent of vascular aging and its associated diseases so that these can then be specifically targeted.

## Einleitung

In Europa ist der Schlaganfall mit 1,1 Mio. Todesfällen pro Jahr die zweithäufigste Todesursache [30]. Der karotisbedingte Schlaganfall macht etwa 15 % der zerebralen Ischämien in Deutschland aus [12]. In der Altersgruppe 60–69 Jahre liegt die Prävalenz einer > 50%igen extrakraniellen Karotisstenose bei 2–2,3 % und steigt bei den > 80-Jährigen auf 5–7,5 % an [12]. Mehr als 80 % der ischämischen Schlaganfälle treten bei Personen über 65 Jahre auf, wobei von diesen fast 25 % über 85 Jahre alt sind [34]. Neben einem aktiven Nikotinabusus, dem männlichen Geschlecht und einer positiven Anamnese für Gefäßerkrankungen ist vor allem das Alter ein Hauptrisikofaktor für das Vorliegen einer Karotisstenose [12].

Das Alter ist ein Hauptrisikofaktor für Karotisstenosen

Eine mögliche Erklärung für das Alter als Risikofaktor liegt in der vaskulären Seneszenz. Hierunter versteht man die graduelle Abnahme struktureller und funktioneller Eigenschaften der Zellen und extrazellulären Matrix der Gefäßwände auf histologischer und zellulärer Ebene. Dies führt zum Verlust des homöostatischen Potenzials und damit zur verminderten Anpassung auf exogene Stressreize [34]. Zu den Mechanismen der vaskulären Seneszenz gehören oxidativer Stress, chronische Entzündung und die mitochondriale Dysfunktion [25].

Nicht nur bei der Entstehung einer Karotisstenose spielt das Altern eine entscheidende Rolle. Das Alter ist auch einer der wichtigsten Risikofaktoren für das perioperative Ergebnis. So ist zum Beispiel die perioperative Schlaganfallrate bei der Stentangioplastie einer symptomatischen Karotisstenose bei älteren im Vergleich zu jüngeren Patienten erhöht, wohingegen dies bei der Karotisendarteriektomie nicht der Fall ist [30]. Als potenzielle Erklärungen hierfür werden eine allgemein erhöhte atherosklerotische Last, vermehrte Kalzifikationen im Aortenbogen, Veränderungen der Gefäßanatomie und eine erhöhte Instabilität der Karotisplaques bei älteren Menschen angesehen [30].

Um neue diagnostische Möglichkeiten und therapeutische Interventionen zur Vorbeugung altersbedingter vaskulärer Pathologien zu entwickeln und eine verbesserte Risikostratifizierung sowie Patientenselektion durchführen zu können, ist das Verständnis der zellulären und molekularen Mechanismen der vaskulären Seneszenz essenziell.

Ziel dieses Übersichtsartikels ist es, den aktuellen Wissensstand über alterungsbedingte Veränderungen von Karotisplaques auf verschiedenen Ebenen aufzuzeigen.

## Altersabhängige Gefäßveränderungen auf multizellulärer Ebene

Ein zunehmendes Alter geht mit morphologischen Veränderungen der Gefäßwand einher, die durch eine Zunahme des Gefäßvolumens und -diameters sowie eine erhöhte Intima-Media-Dicke bestimmt sind. Diesen morphologischen Veränderungen gehen funktionelle Verschlechterungen der Gefäßwand voraus. So ist bekannt, dass die arterielle Steifigkeit und Elastizitätsverlust mit dem Alter zunehmen und sich dadurch die Adaptionsfähigkeit der Gefäße an Blutdruckschwankungen verringert [46]. Es scheint eine lineare Beziehung zwischen der arteriellen Steifigkeit und dem Alter zu bestehen [9], wobei sich die arterielle Versteifung im Alter zwischen 50 und 60 Jahren beschleunigt [10].

Die arterielle Versteifung beschleunigt sich im Alter

Dem liegen atherosklerotische Veränderungen innerhalb der Arterienwand mit Ausbildung typischer atheromatöser Läsionen über folgende Schritte zugrunde: Endothelverletzungen oder Beeinträchtigungen der Endothelschicht führen zu einer Überexpression von Zelladhäsionsmolekülen (CAMs) und anderen Zelloberflächenrezeptoren. Dies erhöht die endotheliale Permeabilität und führt zur Anhäufung von LDL-Molekülen („low density lipoprotein“) in der arteriellen Intima, wo diese leicht oxidiert werden können. Diese oxLDLs fördern wiederum die transendotheliale Migration von Monozyten in die Intima und deren anschließende Differenzierung in Makrophagen. Die infiltrierten Makrophagen produzieren und setzen eine Vielzahl von Zytokinen frei, die im Laufe der Jahre zu einer chronischen Entzündung der Arterienwand führen. Makrophagen reichern sich über die konstitutiv exprimierten Scavenger-Rezeptoren mit oxLDLs an und werden zu Schaumzellen, die die lokale Entzündungsreaktion weiter ankurbeln. Im Laufe der Zeit gehen die Schaumzellen zugrunde und geben die angesammelten Lipide, Triglyzeride und Cholesterin in den extrazellulären Raum ab, die die Plaquebildung einleiten und weiter vorantreiben [33, 40, 41].

**Figure 1:**
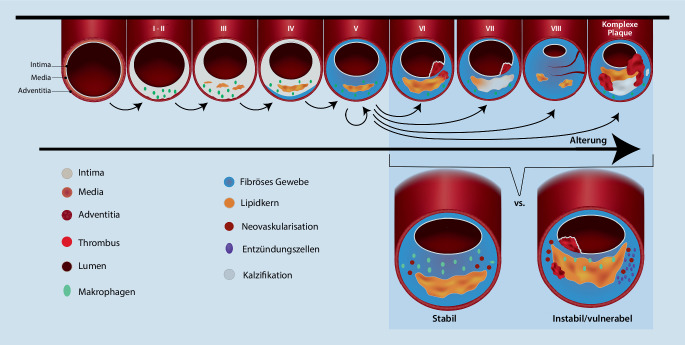

Zu Beginn bleibt die atherosklerotische Läsion stabil mit einer dicken fibrösen Kappe über einer zusammenhängenden nekrotischen Region. Mit zunehmendem Alter und durch verschiedene proatherosklerotische Faktoren wird die fibröse Kappe jedoch dünner, was zu rupturgefährdeten atherosklerotischen Plaques führt ([33, 47]; Abb. 1). Außerdem sind Plaques bei älteren Menschen atheromatöser und weisen aufgrund einer kontinuierlichen Abnahme von Elastinfasern, glatten Muskelzellen und der Gesamtzellularität ein verringertes Bindegewebe auf [33, 34]. Lipidansammlungen, Kalziumablagerungen, Mikroblutungen und mangelhafte Reparaturmechanismen machen die atherosklerotischen Plaques außerdem anfälliger für Verletzungen und daraus resultierende zerebrovaskuläre Ereignisse [33, 41, 47].

## Altersabhängige Gefäßveränderungen auf zellulärer Ebene

Die zelluläre vaskuläre Seneszenz wird durch verschiedene endo- und exogene Stressfaktoren wie reaktive Sauerstoffspezies, Dann-Schäden und eine mitochondriale Dysfunktion verstärkt [42]. Seneszente Zellen wurden bereits mit der Entstehung einer Vielzahl an Krankheiten wie chronisch obstruktiver Lungenerkrankung, Herzversagen, Diabetes mellitus, chronische Niereninsuffizienz, Morbus Alzheimer, Morbus Parkinson, altersbedingte Makuladegeneration sowie Osteoporose und Tumorerkrankungen in Verbindung gebracht [22]. Sie können sich zudem zu einem sog. parakrinen Seneszenz-Phänotyp differenzieren und damit die Funktion benachbarter Zellen beeinträchtigen, was mit zunehmendem Alter zu einer endothelialen Dysfunktion, einer chronischen Entzündung und einem pathologischen Umbau der Arterienwand beiträgt. Die endotheliale Dysfunktion führt zu einer verstärkten Entzündungsreaktion, Thrombose, Gefäßleckage und Beeinträchtigung der antiviralen Immunantwort [23].

Wesentliche Bestanteile der Arterienwand sind Endothelzellen (ECs) und glatte Muskelzellen (SMCs). Unter normalen physiologischen Bedingungen regulieren diese beide Zellarten gemeinsam den systemischen Blutfluss sowie die Immunreaktion, Blutgerinnung und Gewebedurchblutung. Bei älteren Menschen werden ECs flacher und größer und haben einen zunehmend polyploiden Kern [44]. Diese Veränderungen gehen mit der Modulation der Integrität des Zytoskeletts, Proliferation, Angiogenese und Zellmigration einher. Darüber hinaus zeigen seneszente ECs eine erhöhte Freisetzung von Endothelin‑1, eine verminderte endotheliale Produktion von Stickstoffmonoxid sowie die Expression verschiedener Adhäsionsmoleküle und Aktivierung apoptotischer Signalwege [11, 44]. Somit geht die ECs-Seneszenz mit einem Funktionsverlust und proinflammatorischen und proapoptotischen Zustand einher, der die Monozyten-Migration in die Gefäßwand verstärkt und die Atherosklerose weiter fördert. SMCs sind an der Regulierung des Blutdrucks und Gefäßtonus beteiligt [3]. Außerdem sind sie die wichtigsten Zellen für die Synthese verschiedener extrazellulärer Matrixkomponenten zur Stabilisation der Arterienwand. Phänotypwechsel, Apoptose, Nekrose und Transdifferenzierung der SMCs in einen makrophagenähnlichen Phänotyp tragen erheblich zur Gefäßpathologie bei [34]. SMCs aus gealterten Gefäßen zeigen eine verringerte Reaktion auf Mitogene, eine verringerte Proliferationsfähigkeit und eine erhöhte Anfälligkeit für Apoptose. Durch das Zusammenspiel dieser Faktoren ist die Ausbildung einer stabilen fibrösen Kappe auf der arteriosklerotischen Plaque beeinträchtigt, was zu einer weiteren Förderung der Plaqueprogression führt [44].

Seneszente Zellen werden mit vielen Krankheiten in Verbindung gebracht

Die veränderte Expression von Adhäsionsmolekülen auf den seneszenten ECs führt zur weiteren Rekrutierung von Monozyten und deren Differenzierung in Makrophagen innerhalb der Plaques. Die Prozesse begünstigen die Entstehung instabiler Plaques und damit auch das Risiko eines ischämischen Schlaganfalls. Beim Vergleich der Plaquemorphologie verschiedene Altersgruppen konnte bei jüngeren Patienten vermehrt entzündliche Zellinfiltrationen und bei älteren Patienten größere Lipidkerne nachgewiesen wurden. Es konnten jedoch keine signifikanten Unterschiede in der Gesamtinstabilität der Plaques zwischen jungen und alten Patienten festgestellt werden [38].

## Altersabhängige Gefäßveränderungen auf molekularer Ebene

Zu den molekularen Mechanismen der Gefäßalterung gehören oxidativer Stress, eine chronische niedrig-gradige Entzündungsreaktion [42, 44], die mitochondriale Dysfunktion, die Dysregulation verschiedener altersbedingter Faktoren, epigenetische Veränderungen, Autophagie sowie Apoptose und Nekrose [17, 18, 34, 42].

### Theorie der freien Radikale – oxidativer Stress

Eine der am meisten akzeptierten Theorien des Alterns ist die „Theorie der freien Radikale“ [20, 42]. Sie postuliert, dass sich mit dem Alter reaktive Sauerstoffspezies (ROS) ansammeln, was zu oxidativen Schäden an der genomischen DNA, den Proteinen und anderen zellulären Komponenten führt. ROS induzieren die Expression von NF-kB, wodurch verschiedene entzündliche Zytokine wie TNFα, IL6, MCP‑1 freigesetzt werden [42]. Des Weiteren verändern sie die Synthese verschiedener abnormaler Lipidderivate, was in Summe die Atherosklerosebildung weiter fördert [10]. Auch das Renin-Angiotensin-Aldosteron-System (RAAS) ist an der Alterung beteiligt, indem es den oxidativen Stress in den Mitochondrien fördert [8]. Angiotensin II, das wichtigste Effektormolekül des RAASs, aktiviert über seinen Rezeptor die NADPH-Oxidase, die ihrerseits Superoxidanionen (O_2_^−^) erzeugt, was zu einer Beeinträchtigung der Bioverfügbarkeit von Stickstoff (NO) und schließlich zu einer erhöhten ROS-Produktion führt. NO ist der wichtigste Regulator des kardiovaskulären Systems [5]. Eine verringerte NO-Bioverfügbarkeit erleichtert die Adhäsion von Thrombozyten und Leukozyten sowie die Migration und Proliferation von SMCs, was wiederum zu Atherosklerose führt.

### Chronische Entzündungsreaktion im Alter

Mit zunehmendem Alter werden beim Menschen erhöhte Mengen an Entzündungsfaktoren nachgewiesen. Es besteht eine chronische niedriggradige Entzündungsreaktion [37]. Diese ist eine Folge der Immunoseneszenz, die durch altersbedingte unangemessene Reaktionen des angeborenen und adaptiven Immunsystems auf die Exposition von Krankheitserregern und anderen Arten von chronischem Stress charakterisiert ist [16]. Atherosklerose selbst wird als chronische Entzündungserkrankung definiert, bei der die Entzündung alle Phasen des pathogenen Prozesses durchläuft [13]. Proinflammatorische Stimuli und die Überproduktion einer Vielzahl verschiedener Zytokine führen in den Gefäßwänden zur Rekrutierung und Ansammlung von Leukozyten in atherosklerotischen Plaques [26, 45].

Atherosklerose ist eine chronische Entzündungserkrankung

Die verstärkte Präsenz der Immunzellen in der Arterienwand führt zur Freisetzung von inflammatorischen Zytokinen wie IL-1β, INF‑γ, IL‑6, IL‑8 und dem Nox-Signalweg und dadurch zur Induktion von Entzündungsprozessen sowie weiterer ROS-Produktion [50]. Darüber hinaus können ROS die Expression von sog. Scavenger-Rezeptoren auf vaskulären SMCs verstärken und deren Fähigkeit zur Internalisierung und Akkumulation von Lipiden und deren Umwandlung in Schaumzellen induzieren. Die Freisetzung von proteolytischen Enzymen wie Matrix-Metallo-Proteinasen (MMPs) wird ebenfalls durch ROS stimuliert [13]. Des Weiteren nimmt z. B. die Phagozytose-Fähigkeit der Makrophagen mit dem Alter ab, was auf eine Schwächung der mikrobiellen Aktivität hinweist. Auch der Phänotyp und die Funktion von T‑ und B‑Zellen lässt im Alter nach [52]. Dies führt dazu, dass altersbedingte nachteilige Veränderungen der adaptiven Immunantwort häufiger auftreten [32]. Zudem konnten bei älteren Menschen eine verringerte Avidität (Kraft von Antikörpern, eine multivalente Bindung mit einem Antigen einzugehen) und eine quantitative Abnahme der Antikörperreaktion beobachtet werden [42]. Diese Veränderungen schwächen das Immunsystem und begünstigen die bakterielle und virale Infektion im fortgeschrittenen Alter, wie z. B. die COVID-19-Erkrankung, wo SARS-CoV-2-Viren leichter Endothelzellen infizieren können und somit maßgeblich auch den Krankheitsverlauf erschweren [19].

### Epigenetik

Epigenetische Veränderungen sind ein wichtiger Mechanismus der Regulierung von Gen-Stilllegung und -Aktivierung. Altersbedingte epigenetische Veränderungen wurden bereits mit verschiedenen Krankheiten des vorzeitigen Alterns in Verbindung gebracht [17, 18, 48, 49]. Unter anderem verändern sich die DNA- und Histon-Methylierungsmuster in atherosklerotischen Plaques und den korrespondierenden ECs, SMCs und Leukozyten [18]. Eine veränderte Methyltransferase-Expression geht mit der DNA-Hypomethylierung einher, die an Proliferation, Apoptose und Lipidstoffwechsel beteiligt sind [21]. Histonacetylierung und -deacetylierung sind ebenfalls wichtige Faktoren, die zu Atherosklerose und Alterung beitragen [17]. Die Abnahme des altersabhängigen DNA-Methylierungsniveaus geht mit einer hohen Methylierung einiger spezieller Genloci einher, wie zum Beispiel c‑fos, IGF‑2 und p16INK4a. Die Hauptregulatoren des DNA-Methylierungsstatus sind die DNMT3A (DNA-Methyltransferase 3 α) und TET2 („tet methylcytosine dioxygenase 2“). Eine Herunterregulierung dieser trägt zur verminderten Differenzierungsfähigkeit der Zellen bei [6]. Die Hypomethylierung des endothelialen NO-Synthase- (eNOS-)Genpromotors und die Hypermethylierung des Transkriptionsfaktors JunD führen zu endothelialer Dysfunktion und erhöhter ROS-Produktion. Darüber hinaus wird die Hypomethylierung vieler Gene mit Entzündungen, Zelldysfunktion und Gefäßverletzungen in Verbindung gebracht [18, 24]. DNA-Methyltransferasen (DNMTs), Histon-Methyltransferasen (HMTs) und Histon-Acetyltransferasen (HATs) stehen in engem Zusammenhang mit der Umgestaltung des Chromatins und damit der Genexpression.

Deacetylasen, wie die Sirtuine (SIRT) werden als wichtige Regulatoren des Alterungsprozesses angesehen.

Deacetylasen sind wichtige Regulatoren des Alterungsprozesses

Es wird berichtet, dass SIRT1 die Genexpression, den Stoffwechsel und die Alterung durch Deacetylierung einer Reihe von Enzymen und Transkriptionsschaltern wie PGC-1a, NF-kB, eNOS, FOXO, p53, p300/CBP, H3K9 und H3K56 reguliert [24]. Es konnte gezeigt werden, dass eine Überexpression von SIRT1 die Migration von Endothelzellen erhöht, während ein Verlust der SIRT1-Aktivität zu einer Herunterregulierung wichtiger angiogener Faktoren wie Flt1 und CXCR4 führt [36]. SIRT1 verringert zudem den oxidativen Stress und Entzündungen in Gefäßen, indem es NF-kB und PARP hemmt [53]. Darüber hinaus führt eine SIRT1-Downregulation zu einer Histon-Hyperacetylierung, Rekrutierung von Makrophagen und verstärkten Expression von entzündungsfördernden Faktoren wie IL-1b, -6, -4, -10, -14, und TNF‑α, was wiederum Entzündungen begünstigt.

SIRT3 reguliert die Angiogenese durch Erhöhung der VEGF-Expression [24]. Das Histon H3 lysine 9 (H3K9)-Deacetylase SIRT6 hingegen hat eine schützende Wirkung auf seneszente ECs.

### Nichtkodierende RNAs

Nichtkodierende RNAs wie microRNAs (miRs) und insbesondere lange nichtkodierende RNAs (lncRNAs) spielen eine wichtige Rolle bei verschiedenen Prozessen im Gefäßsystem [2, 39]. Sie beeinflussen die Entwicklung, das Wachstum, den Umbau und die Angiogenese, indem sie zelluläre Prozesse wie Proliferation, Apoptose, Adhäsion, Migration und Differenzierung von ECs und SMCs regulieren [39]. So ist beispielsweise die lncRNA des maternal exprimierten Gens 3 (Meg3) in alten menschlichen Herzarterien und seneszenten Endothelzellen hochreguliert [4]. Es wird vermutet, dass Meg3 die miR-21-Expression in ECs unterdrückt und dadurch die Kollagenexpression und Proliferation beeinflusst. Hypoxie, ein wichtiger Auslöser der Angiogenese, erhöht die Expression von Meg3 [29]. Auch Entzündungen, werden nachweislich von Meg3 beeinflusst [2]. Eine weitere nukleäre lncRNA, die durch Hypoxie induziert wird, ist das metastasenassoziierte Lungenadenokarzinom-Transkript 1 (MALAT1), auch bekannt als NEAT2 [43]. Die Expression von MALAT1 ist bei Seneszenz und in atherosklerotischen Plaques reduziert [9]. Es wurde festgestellt, dass MALAT1 an den Transkriptionsfaktor „cAMP response element-binding protein“ (CREB) bindet und dessen Dephosphorylierung durch Proteinphosphatase 2A (PP2A) hemmt, was zu abnormaler Zellviabilität und Hyperproliferation führt.

Bei Atherosklerose und in ECs interagiert MALAT1 mit miR-214 und hemmt die Proliferation und Migration [2]. Ähnlich wie MALAT1 ist die lncRNA MIAT in Leukozyten von Patienten mit ischämischem Schlaganfall hochreguliert. Die Überexpression von MIAT unterdrückt miR-29b, was zu einer verminderten Zelllebensfähigkeit und erhöhter Apoptose führt. Die zytoplasmatische lncRNA H19 zeigt eine erhöhte Expression bei atherosklerotischen Erkrankungen wie zum Beispiel bei Aortenaneurysmen [14]. Genetische Variationen in H19 wurden mit einem erhöhten Risiko für ischämische Schlaganfälle in Verbindung gebracht.

Eine weitere nichtkodierende RNA ist die lncRNA-p21, die in atherosklerotischen Plaques erhöhte Werte aufweist. In SMCs und Makrophagen interagiert lncRNA-p21 mit dem sog. „mouse double minute 2 protein“ (MDM2), wodurch die Apoptose reduziert wird, wohingegen es in ECs die Proliferation hemmt [2]. lncRNAs sind ebenfalls an epigenetischen Modifikationen beteiligt [48]. So induziert H19 verschiedene Modifikationen am Histon H3K9 und damit der transkriptionellen Kontrolle mehrere Gene. Dazu gehört auch das Gen von IGF2, welches an der Entstehung der Atherosklerose beteiligt ist [25]. Darüber hinaus nimmt die Expression der nichtkodierenden Antisense-RNA im INK4-Locus (ANRIL) mit dem Alter zu, was mit Atherosklerose, intrakraniellen Aneurysmen und weiteren kardiovaskulären Erkrankungen in Verbindung gebracht wurde [2]. Der Verlust von ANRIL induziert zudem die zelluläre Seneszenz [7].

## Zukunftsperspektiven

**Figure 2:**
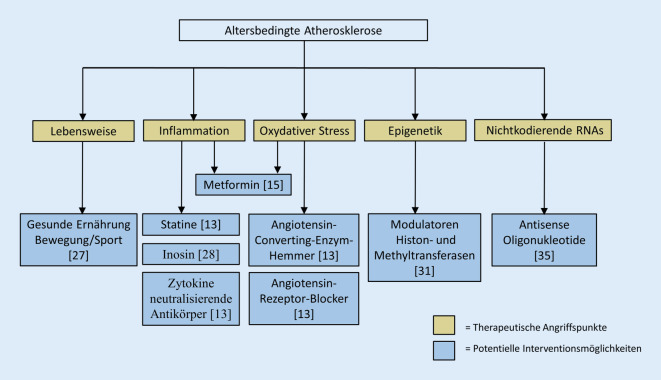

Aufgrund der epidemiologischen Folgen der Atherosklerose wurden große Anstrengungen unternommen, um wirksame Therapeutika zu entwickeln (Abb. 2; [51]). Therapien wie Ernährungsumstellung, Bewegung und die Gabe von Statinen werden bereits erfolgreich eingesetzt [13, 27]. Letztere, sowie die oft gegen arterielle Hypertonie eingesetzten Wirkstoffe Angiotensin-Converting-Enzym-Hemmer und Angiotensin-Rezeptor-Blocker besitzen über ihre Hauptrolle hinaus zusätzliche antioxidative Eigenschaften [13]. Vielversprechende zusätzliche Wirkungen besitzt das Antidiabetikum Metformin. Es beeinflusst die Funktion von Makrophagen, führt zu einer Verringerung der Monozyten-Differenzierung und hemmt dadurch Entzündungsreaktionen, oxidativen Stress, Schaumzellenbildung und Apoptose [15]. Inwieweit jedoch Metformin altersbedingte Gefäßveränderungen vorbeugen kann, gilt es noch überprüfen.

Neuen Arzneimitteln, die der zentralen Pathophysiologie der Entstehung von Atherosklerose entgegenwirken, indem sie ROS abfangen und die Endzündungsreaktion eindämmen, könnte zusätzlich eine entscheidende Bedeutung zukommen [1, 13]. Antikörper, die entzündliche Zytokine (TNF‑α, IL-1β, IL‑6, IL-17 und IL-12/23) neutralisieren, haben vielversprechende, leider aber auch widersprüchliche Ergebnisse gezeigt und bedürfen daher weiterer Forschung [7]. Kürzlich wurden die pharmakologischen Eigenschaften von Inosin entdeckt, einem NF-kB-Modulator, der als potenzielles Medikament zur Behandlung von Herz-Kreislauf-Erkrankungen in Betracht gezogen werden könnte [28].

Was therapeutische Strategien gegen altersbedingte epigenetische Veränderungen betrifft, so deuten präklinische und klinische Studien darauf hin, dass die gezielte Beeinflussung ausgewählter epigenetischer Faktoren eine vielversprechende neue therapeutische Strategie gegen kardiovaskuläre Erkrankungen und damit auch gegen den ischämischen Schlaganfall sein könnte. Die gezielte Beeinflussung beispielsweise von Histondeacetylaten (HDACs) hat Erfolg versprechende Ergebnisse bei der Modulation des Krankheitsverlaufs gezeigt [31]. Durch neuere Entwicklungen auf dem Gebiet der RNA-Therapeutika wird es möglich, nichtkodierende RNAs (insbesondere lncRNAs) als potenzielle Ziele für therapeutische Interventionen zu identifizieren. Die nukleotidbasierte Gentherapie, einschließlich antisense Oligonukleotiden (ASOs) und „small interfering RNAs“ (siRNAs), zeigen dabei vielversprechende Resultate [35].

Zusammengefasst ist die gezielte Bekämpfung von Entzündungen und oxidativem Stress auf medikamentöser und molekularer Ebene eine hoffnungsvolle Strategie zur Behandlung von Atherosklerose und altersbedingten Veränderungen der Gefäße (Abb. 2). Es sind jedoch noch weitere Anstrengungen und Studien erforderlich, um zielgerichtete effizientere antiatherogene Wirkstoffe mit unterschiedlichen Wirkmechanismen zu entwickeln. Es handelt sich bei allen genannten Ansätzen um systemische Behandlungskonzepte der Atherosklerose, womit die Gesamt-Morbidität und -Mortalität gesenkt werden könnte. Ziel ist es dabei aber nicht, eine spezifische Prophylaxe, wie z. B. rein zur Vorbeugung einer Karotisstenose, zu entwickeln.

## Fazit für die Praxis

- Atherosklerotische Plaques der Karotis verändern sich mit zunehmendem Alter. Diese Veränderungen sind multifaktoriell bedingt und hängen neben dem Alter vom individuellen Lebensstil, Komorbiditäten, Umwelteinflüssen und der Genetik des Patienten ab.
- Folgende Veränderungen lassen sich mit zunehmendem Lebensalter in der Plaquezusammensetzung beobachten: Abnahme der Elastinfasern, der glatten Muskelzellen und der Gesamtzellularität, sowie eine Zunahme des Lipidkerns, von Mikroblutungen und Verkalkungen.
- Die altersassoziierte Plaquemorphologie scheint sich in Richtung vulnerabler Plaques zu verändern.
- Weitere Studien sind notwendig, damit in Zukunft durch verbesserte zelluläre und molekulare Analysen die Auswirkungen des Alters auf atherosklerotische Läsionen und das damit einhergehende individuelle kardiovaskuläre Risiko suffizient bestimmt und zielgerichteter behandelt werden kann.
